# The Genetics behind Sulfation: Impact on Airway Remodeling

**DOI:** 10.3390/jpm14030248

**Published:** 2024-02-25

**Authors:** Charikleia Ntenti, Eleni Papakonstantinou, Liana Fidani, Daiana Stolz, Antonis Goulas

**Affiliations:** 1 1st Laboratory of Pharmacology, School of Medicine, Aristotle University of Thessaloniki, 54124 Thessaloniki, Greece; 2 Clinic of Respiratory Medicine, Faculty of Medicine, University of Freiburg, 79106 Freiburg, Germany; 3 Clinic of Respiratory Medicine and Pulmonary Cell Research, University Hospital Basel, 4031 Basel, Switzerland; 4 Laboratory of Medical Biology-Genetics, School of Medicine, Aristotle University of Thessaloniki, 54124 Thessaloniki, Greece; 5 Special Unit for Biomedical Research and Education, School of Medicine, Clinical Research Unit, Aristotle University of Thessaloniki, 54124 Thessaloniki, Greece

**Keywords:** COPD, asthma, sulfatase-modifying factor 1, airway remodeling, sulfatase, single nucleotide polymorphism (SNP)

## Abstract

In COPD, chronic inflammation and exposure to irritants, such as cigarette smoke, lead to the thickening of bronchial walls. This results from increased deposition of collagen and other extracellular matrix components, contributing to the narrowing of airways. Nevertheless, it is widely recognized that COPD is an inflammatory disorder marked by partially reversible airflow limitation wherein genetic factors interact with the environment. In recent years, numerous investigations have substantiated the correlation between gene polymorphisms and COPD. *SUMF1* has been implicated in diverse cellular processes, including lysosomal function and extracellular matrix maintenance, both of which play pivotal roles in respiratory health. The genetic variations in *SUMF1* could lead to an imbalanced sulfation in the extracellular matrix of lung tissue, potentially playing a role in the onset of COPD. Recent studies have uncovered a potential link between dysregulation of *SUMF1* and COPD progression, shedding light on its involvement in the abnormal sulfatase activity observed in COPD patients. Through a comprehensive review of current literature and experimental findings, this article aims to contribute to the growing body of knowledge surrounding the genetic intricacies concerning sulfation of airway remodeling and possible pharmacological applications in COPD and asthma management.

## 1. Introduction

Asthma and chronic obstructive pulmonary disease (COPD) are defined by the presence of airflow obstruction, measurable structural changes in the airways [1,2], and inflammation [3,4].

According to the World Health Organization, chronic obstructive pulmonary disease (COPD) is anticipated to rank as the fourth leading cause of global mortality by 2040 [5].

COPD is characterized by chronic bronchitis, remodeling of small airways, and the presence of emphysema, predominantly affecting the elderly as a disease associated with accelerated lung aging [6]. Emphysema’s hallmark feature involves the breakdown of alveolar structures, resulting in enlarged air spaces and a diminished surface area. Existing experimental evidence proposes that the development of emphysema is propelled by the expedited senescence of lung cells; nevertheless, the precise mechanism underlying this senescence process remains elusive [7]. COPD is predominantly associated with prolonged cigarette smoking [8], yet diverse genetic factors can influence susceptibility to lung damage and the subsequent development of COPD [9]. These factors range from genetic, infectious, and inflammatory factors to social aspects linked to lifestyle modifications. Much of the knowledge regarding the pathological features of COPD is derived from studies on smokers, revealing an intensified inflammatory response to chronic irritants and structural alterations resulting from repetitive injury and healing processes, which persist even after smoking cessation. In non-smokers, additional factors such as systemic inflammation, genetic predisposition, oxidative stress, and alterations in the lung microbiome may also contribute to the disease. COPD stands as a persistent inflammatory lung condition, with inflammation playing a pivotal role in the initial stages of emphysema, particularly concerning the mechanisms triggering early alveolar destruction [10,11].

On the other hand, asthma also exhibits high prevalence, impacting a total of 262 million individuals, as reported by the World Health Organization (WHO). The primary form of asthma, known as atopic asthma, is induced by allergic inflammation triggered by allergens, leading to tissue damage. This damage is induced by eosinophils’ major basic protein [12], proteases of mast cells [13], or neutrophil extracellular traps in cases of inflammation associated with neutrophils [14]. If the inflammatory response is brief, repair processes can eliminate the damage. However, persistent inflammation leads to excessive repair processes, causing the remodeling of lung tissue. This remodeling includes hyperplasia of mucus-producing goblet cells, thickening of the basal lamina of the airway epithelium, neovascularization, and the formation of fibrotic foci below the basement membrane [15]. Taken together, these processes contribute to airway obstruction [16]. Consequently, it is widely acknowledged that cellular senescence and inflammation are intricately linked in the context of accelerated or premature lung aging, often referred to as “inflammaging” [12,17,18]. 

Due to the heterogeneous nature of COPD, patients likely exhibit diverse genetic patterns. The decline in lung function observed in COPD results primarily from airway inflammation induced by oxidative stress, leading to airway remodeling and tissue degradation. It is crucial to explore different gene polymorphisms associated with these processes to gain insights into their roles in disease progression. Alpha-1 antitrypsin deficiency, linked to the *SERPINA1* gene polymorphism, stands out as a well-known genetic factor in developing COPD [19]. Additionally, various single nucleotide polymorphisms (SNPs) associated with inflammatory processes and biological stress pathways have been identified in connection with COPD [20,21]. Polymorphisms related to connective tissue remodeling, such as matrix metalloproteinase (MMP)-7 influencing early COPD development [22] and MMP-12 associated with severe/very severe COPD, have also been recognized [23]. A shared pathological characteristic among the two diseases is fibrotic tissue mainly present in the airway wall.

## 2. Alveolar Formation

Alveolarization is a complex process influenced by the intricate interplay of extracellular matrix proteins (ECM) and secreted growth factors [24]. ECM, as a three-dimensional scaffold present in the airways, offers physical support to cells and provides biochemical signals crucial for cellular processes such as morphogenesis, differentiation, and tissue homeostasis [25]. The alveolar ECM consists of various components, including type IV collagen, laminin, entactin/nidogen, tenascin, integrins, elastin, fibrillins, and proteoglycans [26]. Proteoglycans (PG) are structured with a protein core to which one or more highly sulfated polysaccharide chains, known as glycosaminoglycans (GAGs), attach. Comprising macromolecules, primarily structural proteins and GAGs, the ECM includes polysaccharides like galactosamine, N-acetylgalactosamine-4-sulphate, galactose, or galacturonic acid in repeating disaccharide units. Major GAG types in the airways and lungs, such as heparin/heparin sulfate, chondroitin sulfate, dermatan sulfate, hyaluronic acid, and keratin sulfate, are typically covalently linked to core proteins like syndecan (chondroitin and heparin sulfate), decorin (dermatan sulfate), and glypican (heparan sulfate) [27]. These proteoglycans play a crucial role in stabilizing the three-dimensional fibrillar matrix, providing resistance to tissue compression and accommodating interstitial fluid expansion. Additionally, the glycosaminoglycans within proteoglycans have been recognized for regulating various functions in organ growth, as well as cell differentiation and proliferation [28]. The ability of glycosaminoglycans to mediate these functions is contingent on their degree of sulfation, a characteristic determined by sulfotransferases that incorporate sulfated groups and sulfatases that remove them [29,30]. In the early stages of human lung development, collagens I, III, and VI, along with proteoglycans (decorin, biglycan, and lumican), are primarily observed at the interface between the epithelium and the mesenchyme, forming a sleeve around the developing airways [31]. The proteoglycan component of the ECM may play a role in regulating airway branching, partly due to the capacity of sulfated proteoglycans to bind with FGF10, a crucial factor for branching [32]. Moreover, GAGs within the ECM interact with various proteins, including chemokines, cytokines, and adhesion molecules [33]. Due to their common association with the cell membrane, GAGs function as cell surface receptors or co-receptors, capturing ligands necessary for activating downstream signaling. For instance, heparan sulfate (HS) and its proteoglycan syndecan play a role in capturing the fibroblast growth factor (FGF) receptor (FGFR), facilitating its internalization and endosomal sorting in an FGF-dependent manner [34]. Cell surface GAGs also bind to chemokines released during tissue injury, guiding leukocyte migration, and promoting inflammation, potentially influencing tissue repair or healing [35]. 

## 3. Sulfatases Pivotal in Extracellular Matrix Remodeling

Previous research has indicated that there is a compromise in heparan sulfate in emphysema, potentially resulting in significant disruptions in the coordination of growth factors, proteases, and other ECM molecules [36]. This disruption may lead to hindered lung tissue repair and regeneration, ultimately contributing to the development of emphysematous lesions [37]. Additionally, chondroitin sulfate has been demonstrated to play a dynamic role in the growth and morphogenesis of the embryonic lung [38]. Dermatan sulfate, on the other hand, impacts the function of growth factors within the lung and influences proliferation in a cell-type-specific manner. Moreover, dermatan sulfate serves as a docking molecule for the adhesion of various human pathogenic microorganisms [39]. 

Sulfated proteoglycans (PGs) are composed of core proteins that undergo covalent modification through the addition of sulfate chains, featuring variably sulfated repeating disaccharide units [38]. These molecules play a crucial role in numerous signaling functions, utilizing their sulfated chains to bind diverse protein ligands, including growth factors, morphogens, chemokines, and cytokines. The effectiveness of these ligand interactions is heavily influenced by the pattern and density of sulfation modifications, with particular emphasis on the significance of 6-O-sulfation of glucosamine (6OS) in many interactions [40]. 

A unique regulatory mechanism for PG-dependent signaling is introduced by two extracellular sulfatases, namely SULF1 and SULF2. These enzymes operate at neutral pH and function extracellularly to remove 6OS from intact PGs. This removal by SULFs presents a novel means of influencing PG-dependent signaling. Notably, the enzymatic activity of SULFs contributes to the modulation of key signaling pathways by mobilizing protein ligands (such as Wnt, GDNF, PDGF-B, and BMP-4) from PGs’ sequestration. Consequently, this liberation enables the ligands to bind to signal transduction receptors, facilitating downstream signaling events. SULF1 and SULF2 sulfatases in humans are processed by furin-like endoproteases and form disulfide-bond-linked heterodimers of 75 kDa and 50 kDa. This is a key event in the translocation of SULFs in specialized membrane microdomains. Both SULF1 and SULF2 take out sulfate groups on C-6 positions of glucosamines, trisulfated disaccharides of heparin/heparan sulfate (HS) glycosaminoglycan chains [41,42]. Sulfatases play a vital role in the intricate equilibrium of connective tissue remodeling by eliminating sulfate from specific sulfated carbohydrate chains. SULFs have been recently identified as members of the sulfatases’ family. A core event in the characterization of sulfatases is the discovery of QSulf-1 in quail, and then the discovery of their orthologs in rodents and humans [43,44]. Following the discovery of QSulf-1, the cloning and characterization of *Sulf-1* took place. It is of great importance for tissue connectivity and remodeling that, even though most of the known and understood sulfatases are localized in the lysosomes, SULFs are anchored on the cell surface or secreted in the extracellular space. Each SULF molecule contains a signal peptide and two distinct sulfatase-related domains, interrupted by a large hydrophobic domain. Successful SULFs activation requires post-translational modification with formylglycine and N-linked glycans [43,45]. Therefore, the SULFs are endosulfatases that remove sulfate esters from glycosamine inside the appropriate contexts of heparin and HS chains [42]. SULF2, a 6-O sulfatase of the extracellular matrix (ECM), desulfates heparan sulfate proteoglycans (HSPGs), releasing growth factors and cytokines from storage sites and activating downstream signaling pathways, including FGF, VEGF, PDGF, IL-6, TGFβ, and WNT [46]. SULF2 influences fibrotic liver disease by interacting with the TGF-β1/Smad pathway, and it appears that Transforming Growth Factor Beta Receptor 3 (TGFBR3) plays a significant role in mediating the activation of the TGF-β1 signaling pathway by SULF2 [47]. While variations in the *SULF2* gene have been documented and linked to the pathogenesis of several diseases, there is currently no reported association with COPD or other lung diseases.

Although the exact importance of fibrosis-related alterations in the sulfation profile remains uncertain, alterations in the sulfation of Chondroitin sulfate (CS) and Dermatan sulfate (DS) chains might be associated with the processes involved in ECM remodeling during lung injury and repair in lung fibrosis. Elevated sulfation levels could create new epitopes by introducing additional negative charges, imparting novel physical and chemical characteristics to GAG chains. Furthermore, it is plausible that changes in the sulfation pattern of CS/DS GAG chains may lead to an increased affinity for growth factors, adhesion molecules, or other cytokines, potentially aiding in matrix repair by fibroblasts and/or myofibroblasts. Changes in the expression of GAGs and their sulfation patterns may also impact several biologically significant events, such as the accumulation of inflammatory cells, cellular adhesion, migration, and proliferation. Selective desulfation of endogenous HS and inhibition of HS biosynthesis have been shown to increase cellular iron in cell lines and mice, respectively [48]. 

## 4. Sulfatase Modifying Factor 1—The Master Regulator of Sulfatases Activity

**Figure 1:**
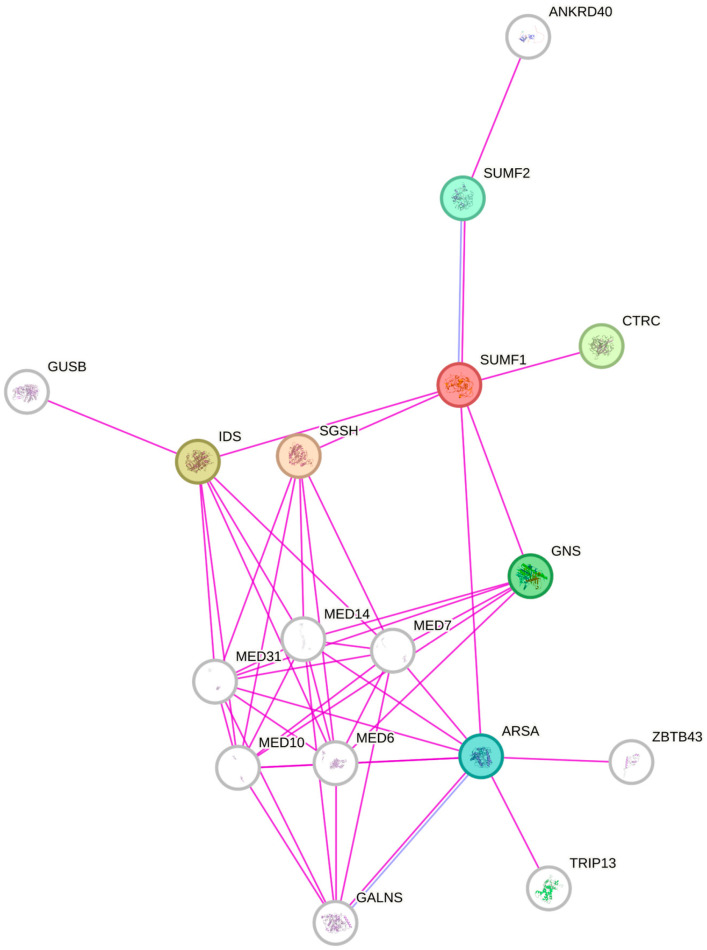
Protein–protein interaction (PPI) diagram. STRING database (https://string-db.org, assessed on 10 February 2024) was used to identify the interactions between the molecules. The pink lines represent experimentally determined interactions among the proteins. SUMF1: Formylglycine-generating enzyme; Oxidase that catalyzes the conversion of cysteine to 3-oxoalanine on target proteins, using molecular oxygen and an unidentified reducing agent. 3-oxoalanine modification, which is also named formylglycine (fGly), occurs in the maturation of arylsulfatases and some alkaline phosphatases that use the hydrated form of 3-oxoalanine as a catalytic nucleophile. Known substrates include GALNS, ARSA, STS and ARSE. Belongs to the sulfatase-modifying factor family. (374 aa). SUMF2: Inactive C-alpha-formylglycine-generating enzyme 2; Lacks formylglycine generating activity and is unable to convert newly synthe. GNS: N-acetylglucosamine-6-sulfatase; Glucosamine-6-sulfatase; Belongs to the sulfatase family. ARSA: Arylsulfatase A component B; Hydrolyzes cerebroside sulfate. CTRC: Chymotrypsin-C; Regulates activation and degradation of trypsinogens and procarboxypeptidases by targeting specific cleavage s SGSH: N-sulphoglucosamine sulphohydrolase; Catalyzes a step in lysosomal heparan sulfate degradation. Belongs to the sulfatase family IDS: Iduronate 2-sulfatase 14 kDa chain; Lysosomal enzyme involved in the degradation pathway of dermatan sulfate and heparan sulfate.

To initiate the hydrolysis of their natural substrates, sulfatases require post-translational activation. A consensus sequence within their catalytic domain includes a cysteine that undergoes modification into formylglycine (FGly), a process facilitated by the formylglycine-generating enzyme encoded by the sulfatase modifying factor 1 (*SUMF1*) gene [49,50,51]. *SUMF1*, the key controller for all recognized sulfatases in the organism, transforming them into an active state, a protein-coding gene, encodes the formylglycine-generating enzyme (FGE) responsible for the conversion of cysteine to FGly [52]. This enzyme facilitates the breakdown of sulfate esters by causing the oxidation of a cysteine residue within the sulfatase substrate, leading to the formation of an active site 3-oxoalanine residue. In the lungs, GAGs are distributed within the ECM [53]. SUMF1 carries out a unique post-translational modification essential for sulfatase activity, allowing it to desulfate GAGs [30,54]. *SUMF1* carries out its function within the endoplasmic reticulum (ER) where it activates all newly synthesized sulfatases (Figure 1); however, it also has the ability to be secreted and taken up by distant cells and tissues, where it relocates within the ER as an active enzyme [55]. 

Multiple sulfatase deficiency (MSD) is a monogenic disorder in humans characterized by simultaneous defects in all sulfatase activity [49]. Individuals with MSD exhibit mutations in the *SUMF1* gene [49]. As a mouse model of MSD, a *Sumf1*^−/−^ strain has been developed, displaying a complete loss of sulfatase activities, early mortality, congenital growth retardation, skeletal abnormalities, neurological defects, and a generalized inflammatory process affecting various organs [56]. The emphysema-like characteristics observed earlier suggested a potential involvement of *SUMF1* in the onset of COPD, characterized by emphysema development [57]. Apart from the emphysema-like features, extensive accumulation of GAGs was identified in various cell and tissue types in SUMF1^−/−^ mice [58]. In a preceding study, several SNPs affecting the gene expression of specific splice variants in *SUMF1* were discovered and associated with COPD [59]. 

Mutations in *SUMF1* stand as the leading cause of various human diseases, many of which have detrimental effects on the lungs [59]. Individuals with impaired SUMF1 activity experience an accumulation of sulfated GAGs, resulting in multiple sulfatase deficiency, a disorder characterized by lysosomal storage issues. The human genome encodes a total of 17 sulfatases, with 13 of them biochemically characterized. Two decades ago, the number of identified sulfatases was quite limited and unable to explain the vast diversity of sulfated molecules. At least five of the known sulfatases are essential for non-redundant desulfation of GAGs in the lysosome. Dysfunction in sulfatase activity, particularly in multiple sulfatase deficiency, results in intralysosomal storage and cellular damage.

On one hand, a *SUMF1* mutation contributes to a decline in pulmonary function by impacting alveolar function. The coordination of alveolar formation, also known as alveolization, involves intricate regulation and complex interactions between growth factors and extracellular matrix proteins [60]. It has been confirmed that sulfatase cannot achieve full activation in SUMF1 (^−/−^) mice. This leads to the deposition of highly sulfated GAGs in the alveoli, reducing the alveolar septum and increasing alveolar volume, ultimately resulting in decreased lung function [58]. 

Despite its well-established role in multiple sulfatase deficiency [61], observations by Arteaga-Solis et al. [58] revealed that Sumf1^−/−^ mice displayed a lung phenotype akin to emphysema, linked to a post-natal arrest in alveolarization. In a previous clinical investigation, several single nucleotide polymorphisms (SNPs) in *SUMF1*, particularly rs793391, exhibited notable associations with COPD. Sumf1^−/−^ mice exhibited a lung phenotype resembling emphysema, attributed to post-natal alveolarization arrest. In a prior clinical study, several SNPs in SUMF1, notably rs793391, showed significant associations with COPD.

Twelve Single Nucleotide Polymorphisms (SNPs) in the *SUMF1* gene were identified as noteworthy through the analysis of expression quantitative trait loci (eQTL). Certain variations in the splicing patterns of *SUMF1* demonstrated diminished expression levels in sputum cells among individuals with chronic obstructive pulmonary disease (COPD) in comparison to those in the control group [59]. Moreover, when considering the *SUMF1* SNP rs11915920 in conjunction with the expression quantitative trait loci (eQTL) analyses, it emerged as a prominent genetic marker. Reduced mRNA expression levels were noted in sputum cells and lung fibroblasts among individuals with the variant allele, aligning and confirming the outcomes of the eQTL analysis conducted on lung tissue. The findings confirm and align with the results of the lung tissue eQTL analysis [61]. Notably, a previous genome-wide association study identified an association between SUMF1 and prominent emphysema, although this association was not further investigated [62]. The role of lysosomal sulfatases ARSA, -G, and -K in extracellular matrix remodeling, particularly in COPD, remains unclear, despite their categorization in various lysosomal storage disorders linked to sulfatase deficiency or alterations [54,63,64]. 

*SUMF2* is a paralog gene of the *SUMF1*, representing an additional level of control of the sulfatase activity and sharing a 48% amino-acid identity and 62% similarity with *SUMF1*. Data from the northern blotting analysis showed that the expression of *SUMF2* mRNA is of comparable amount with respect to the *SUMF1* transcript [46]. The similarity on expression levels of SUMF1 and *SUMF2* suggest that these two genes may be coregulated on a transcription level [46]. Furthermore, experiments on cell lines showed that the transcription of *SUMF2* is dependent on *SUMF1* [46].

Despite the advances made in researching the metabolic functions of pFGE (SUMF2 expression) over the past decade, the precise role of SUMF2 in allergic inflammation in asthma remains unclear [55,65,66,67,68,69,70]. Recent investigations by other research groups have indicated that SUMF2 (along with SUMF1) resides in the luminal space of the endoplasmic reticulum (ER), where synthesized sulfatases undergo post-translational modification through the formation of FGly. SUMF2 has the ability to bind to SUMF1 and sulfatase, thereby regulating their activities and the FGly formation process [55,69,70]. Notably, human IL-13 typically undergoes post-translational modification in the ER [71]. Based on these findings, someone could hypothesize that SUMF2 may alter the modification and secretion of IL-13 within the ER, and thus, variations in this gene could have a significant impact on downstream processes.

The most abundant sulfated glycosaminoglycan (GAG) in the lung is heparan sulfate (HS), but chondroitin sulfate (CS) and dermatan sulfate (DS) are also present. HS binds to perlecan, glypicans, and syndecans, playing a vital role in various physiological processes, ECM interactions, and activation of molecules. Chondroitin sulfate (CS) and dermatan sulfate (DS) have crucial roles in ECM protein activation and degradation. Given the impact of GAG sulfation on iron homeostasis, *SUMF1* polymorphisms may influence iron homeostasis in the lung, potentially affecting susceptibility to COPD and/or its progression. The specific direction of this effect is challenging to predict due to limited data on the interaction between sulfated GAGs and BMP6 on BMP6 receptor (BMP6R) functionality. However, patients homozygous for the reference (A) allele of rs793391 displayed lower FEV1/FVC and FEV1% predicted values, suggesting a potential impact on BMP6 signaling and increased hepcidin expression, leading to enhanced iron loading into cells and potential consequences for alveolar architecture [59]. 

## 5. Discussion

In the pathogenesis of COPD, thickening of airway walls due to chronic inflammation is a significant event. Neutrophils, macrophages, and T-lymphocytes participate in the inflammatory process, leading to airway thickening related to the hyperplasia of airway smooth muscle cells and (myo-)fibroblasts and the expanded deposition of extracellular matrix [4,72]. Both bronchial epithelium and airway smooth muscle are involved in airway remodeling in COPD. Cytokines and growth factors, such as platelet-derived growth factor-B (PDGF-B), epidermal growth factor (EGF), and transforming growth factor-β (TGF-β), play a significant role in this remodeling, released from the sites of the airway wall (Remodeling in Asthma and Chronic Obstructive Lung Disease, n.d.). Fibroblast growth factors (FGFs) have an important role in the regulation of airway remodeling, with members of the EGF and FGF family playing a part in persistent inflammation and tissue repair processes, leading to pulmonary fibrosis [73,74]. According to these data, cell–cell interactions and interactions between cells and various growth factors including basic FGF (FGF-2), insulin-like growth factor-1 (IGF-1), PDGF-B, TGF-β, endothelin-1 (ET-1), and EGF, result in enhanced cellular proliferation and increased collagen expression [75]. 

The FGF–FGFR system is hypothesized to be involved in the pathogenesis of COPD, with expression profiles of FGF-1, FGF-2, and FGFR-1 analyzed in bronchial airways of individuals who are current or former smokers, with or without COPD [76]. The findings of the study revealed an elevation in the bronchial expression of FGF-1, FGF-2, and FGFR-1 in COPD patients, suggesting their potential involvement in regulating pulmonary airway remodeling [77]. 

Within the lung, the extracellular matrix (ECM) assumes a vital role in both the appropriate construction and upkeep of alveolar structure, underscoring the significance of proteoglycans in lung architecture. The pulmonary extracellular matrix holds sway over the tissue architecture of the lung, ensuring the essential mechanical stability and elastic recoil required for normal physiological lung function [78]. 

Although the significance of fibrosis-related changes in the sulfation profile is presently unknown, aberrations in the sulfation of CS/DS chains may be linked to the processes of ECM remodeling during lung injury and repair in pulmonary fibrosis. Increases in sulfation may generate neoepitopes by adding more negative charges and may bestow novel physical and chemical properties to GAG chains. In addition, it is feasible that changes in sulfation pattern of CS/DS GAG chains may result in increased affinity for growth factors, adhesion molecule, or other cytokines that may assist in matrix repair by fibroblasts and/or myofibroblasts [79].

Alterations in expression of GAGs and their sulfation pattern could also affect at least some biologically significant events, such as inflammatory cell accumulation, cellular adhesion, migration, and proliferation [80,81,82,83,84]. *SUMF1* exhibits distinct expression patterns in sputum cells obtained from both COPD patients and controls. Studies unveiled certain SNPs in the *SUMF1* gene that markedly influence mRNA levels, as evidenced by an expression quantitative trait loci (eQTL) analysis conducted on a lung tissue dataset. This finding was further validated through in vitro mRNA expression analyses performed on sputum cells and lung fibroblasts. Moreover, specific *SUMF1* SNPs were identified to be linked to an elevated risk of COPD. Notably, these *SUMF1* SNPs demonstrated divergent effects within the context of COPD. For instance, rs11915920 exerted an impact on *SUMF1* mRNA expression across tissue, sputum cells, and lung fibroblasts, while the SNP rs793391 exhibited a significant association with lung function parameters, thereby implicating its role in COPD [59]. It is worth mentioning that rs793391 was not only associated with COPD in smoking/ex-smoking subjects, but also in never-smokers [61]. 

Furthermore, the significance of GAGs in respiratory disease has been underscored by the COVID-19 pandemic. GAGs play a role in regulating the distribution and activity of growth factors based on their degree of sulfation. When SUMF1 is mutated, highly sulfated GAGs promote the signaling of growth factor β (TGF-β), and an upregulation of TGF-β signaling has been observed in SUMF1 (^−/−^) mice.

Recently, *SUMF1* was associated with the outcomes of SARS-CoV-2 infection with the rs794185 polymorphism in the *SUMF1* gene being associated with the severity of COVID-19. The risk of severe COVID-19 at the rs794185 site of the *SUMF1* gene was significantly reduced for TT carriers, while patients possessing at least one C allele at the rs794185 exhibited a reduced likelihood of experiencing severe COVID-19. This may be related to alveolar injury, systemic immune response, and nervous system damage caused by infection [85]. 

This leads to a developmental arrest in alveolar formation, reducing lung function. Transgenic mice overexpressing TGF-β between postnatal days 7 and 14 exhibited bronchopulmonary dysplastic-like lungs due to the suppression of alveolar septation. Similar results were observed in neonatal rats overexpressing TGF-β.

*SUMF2*, a member of the formylglycine-generating enzyme (FGE) family, plays a crucial role in catalyzing the oxidation of a specific cysteine to Cα-formylglycine [65]. Despite considerable progress in understanding the metabolic functions of pFGE (*SUMF2* expression) in the past decade [55,69,70], its involvement in allergic inflammation in asthma remains poorly elucidated. Recent research by other groups has indicated the localization of SUMF2 (and SUMF1) in the luminal space of the endoplasmic reticulum (ER), where synthesized sulfatases undergo post-translational modification through FGly formation. SUMF2 interacts with SUMF1 and sulfatases, influencing their activities and the FGly formation process [55,69]. Notably, human IL-13 typically undergoes post-translational modification in the ER [86] that SUMF2 may impact the modification and secretion of IL-13 in the ER [71]. 

## 6. Conclusions—Future Directions

Asthma and COPD are respiratory conditions that pose significant public health challenges. Despite their distinct nature, both diseases share common remodeling features, albeit manifesting differently in each pathology. Airway remodeling denotes the alterations in both structure and function of airway tissue resulting from persistent chronic inflammatory stimulation, leading to incomplete repair. This phenomenon is prevalent in chronic airway inflammatory conditions like asthma and COPD. It stands as a major contributor to irreversible airway narrowing and restriction of airflow. Numerous studies have delved into the examination of related proteins, both at systemic and local levels, shedding light on these intricate processes. While research into the molecular mechanisms and clinical aspects of airway remodeling has advanced swiftly in recent years, diseases primarily characterized by such remodeling, such as asthma and COPD, still exhibit high rates of prevalence, disability, and mortality.

For this reason, to understand the regulatory mechanisms that lead to the expression of remodeling-related gene products as well as the research studies which analyze the genetic variations and their relationship with the phenotype expressed, it is vital to differentiate the genetic and molecular mechanism of both illnesses and to provide more effective treatment alternatives that contribute to the improvement of the patient.

SNP databases serve as a robust tool for association studies aiming to elucidate connections between a phenotype and specific genomic regions. SUMF1 serves as the master regulator of sulfatase activity in the cell, and polymorphisms in its corresponding gene, *SUMF1*, have been associated with COPD, affecting the gene expression of specific splice variants [80]. Furthermore, research data indicate potential implications of SUMF1 as a therapeutic target for COPD, exploring the prospect of modulating its activity to mitigate disease severity. It is important to independently confirm the link between *SUMF1* polymorphisms with both the occurrence and advancement of COPD. Exploring their potential relevance to asthma pathogenesis is also important. Should these associations prove robust, they may serve as valuable biomarkers for identifying patients who could potentially benefit from personalized prognosis and therapy. Understanding the involvement of *SUMF1* in COPD pathogenesis may pave the way for innovative therapeutic strategies and targeted interventions, offering new avenues for the development of treatments that address the underlying molecular mechanisms of this debilitating respiratory condition.

## Data Availability

Not applicable.
